# Nivolumab in Renal Cell Carcinoma: Current Trends and Future Perspectives

**DOI:** 10.15586/jkcvhl.2018.102

**Published:** 2018-02-06

**Authors:** Cesar E. Ochoa, Richard W. Joseph

**Affiliations:** Division of Hematology/Oncology, Department of Medicine, Mayo Clinic, Jacksonville, FL, USA

**Keywords:** checkpoint inhibitors, immunotherapy, nivolumab, renal cell carcinoma, targeted therapy

## Abstract

Targeted agents form the backbone of most therapeutic strategies in advanced renal cell carcinoma (aRCC) but ultimately resistance develops and toxicity often leads to discontinuation of treatment, limiting the clinical benefits of these treatments. Nivolumab, a fully human IgG4 anti-PD-1 antibody, selectively blocks the interaction between PD-1 and its ligands PD-L1 and PD-L2 and provides a novel therapy option for patients with aRCC. In 2015, the pivotal phase III study CheckMate 025 led to the Food and Drug Administration approval of nivolumab in patients with aRCC who had received prior anti-angiogenic therapy, and in 2017, the phase III study CheckMate 214 showed that combined immunotherapy with nivolumab plus ipilimumab resulted in greater objective response rate and prolonged progression-free survival when compared with sunitinib in intermediate- and poor-risk patients with previously untreated aRCC. Early studies of nivolumab in association with anti-angiogenic therapy have generated enthusiasm and multiple combination trials are ongoing.

## Introduction

Renal cell carcinoma (RCC) represents the most common type of kidney cancer in adults. The global incidence is approximately 337,000 cases with 143,000 patients dying of the disease each year. In 2016, there were an estimated 62,700 cases and 12,200 deaths from RCC in the United States alone (1).

In recent years, treatment options have improved significantly with the introduction of multiple agents that target vascular endothelial growth factor (VEGF) or mammalian target of rapamycin (mTOR). These drugs have been approved based on improvements in progression-free survival (PFS) and overall survival (OS). In November 2015, the US Food and Drug Administration (FDA) approved nivolumab for patients with aRCC who have received prior anti-angiogenic therapy after showing a statistically significant and clinically meaningful difference in OS (2). Nivolumab is a PD-1 checkpoint inhibitor that selectively blocks the interaction between the PD-1 expressed on activated T-cells and its two ligands, PD-L1 and PD-L2. By disrupting the PD-1/PDL1 signaling, nivolumab is thought to restore the immune response and antitumor activity (3). In this mini review, we discuss practical considerations for the use of nivolumab in aRCC.

## Brief History of Immunotherapy in RCC with Interleukin 2 (IL-2) and Interferon-Alpha (IFN-α)

In the late 19th century, William Coley, haunted by the death of a patient with metastatic sarcoma, found 47 case reports in which concomitant infection seemed to cause the remission of an otherwise incurable malignancy. By 1891, Coley began injecting his patient’s tumors with heat-killed *Streptococcus pyogenes* and *Serratia marcescens*, and achieved durable complete remissions in several different types of malignancies (4). Since that time, with exponential advances in the understanding of the immune system, the idea of immunotherapy has returned to prominence.

Several observations, including the regression of metastases observed in RCC patients after nephrectomy, suggested a relationship between RCC and the immune system. These observations, together with disappointing results from treatment with cytotoxic chemotherapy (5), and the recognition of several immunological dysfunctions in patients with RCC (6), generated significant interest in the development and clinical application of immunotherapy.

IL-2 was first identified in 1976 as a T-cell growth factor; it mediates its biologic effect by binding to the IL-2 receptor on activated T-cells promoting the clonal expansion of antigen-specific cells and plays an important role in potentiating the cytotoxic activity of lymphocytes (7). In the 1980s, a series of reports from the National Cancer Institute (8) demonstrated that recombinant IL-2 was capable of mediating the regression of tumors in patients with metastatic or unresectable malignancies that had previously not responded to conventional treatment.

The pooled results of seven phase II studies led to the FDA approval of IL-2 therapy in 1992. The reported overall response rate was 15% and the complete response rate was 7%. The responses were durable, with a median duration of 54 months. Among the patients who achieved a complete response, 83% remained free of recurrence at last follow-up (9, 10). Subsequent large randomized trials continued to show response rates of approximately 20% but failed to demonstrate an OS advantage (11, 12).

IL-2 is a strong stimulator of proinflammatory cytokines and is associated with severe toxicity such as capillary leak syndrome, cardiac arrhythmias, renal failure, and life-threatening skin reactions. Despite the remarkable responses seen in a minority of patients, the need for hospitalization at expert immunotherapy centers to manage the side effects of its administration significantly limited its utilization (13).

A second cytokine that is widely used in the treatment of RCC is IFN-α, a pleotrophic protein with antiviral, immunomodulatory, and antiproliferative activities. In 1983, successful treatment of aRCC with IFN-α (14) provided the basis for multiple phase II studies using human lymphoblastoid interferon and the development of recombinant interferons. The activity of IFN-α in aRCC was later evaluated in a variety of large trials. These studies include comparisons with noncytokine-containing and other cytokine-containing regimens. The overall response rate ranges between 10 and 15% (15). The median improvement of OS was 3.8 months in a meta-analysis of four studies that included 644 patients (16).

Combinations of IFN-α with IL-2, bevacizumab, or chemotherapy did not result in substantial further clinical benefits and therefore immunotherapy with IL-2 or IFN-α was considered to be the standard of care for aRCC despite modest improvements in survival until 2005 when sorafenib, the first orally active multikinase inhibitor, received FDA approval. Since then, targeted agents have been the backbone of most therapeutic strategies in aRCC with objective response rates ranging from 30 to 47% in untreated patients, and from 1.8 to 23% in pretreated patients (17–20).

Ultimately, resistance to targeted therapy develops and toxicity often leads to discontinuation of treatment, limiting the clinical benefits of these treatments (21).

Nivolumab provides a novel therapy option for patients with aRCC. Its mechanism of action differs from other targeted therapies. It is a fully human IgG4 anti-PD-1 antibody that selectively blocks the interaction between PD-1 and its ligands PD-L1 and PD-L2 restoring the anticancer immune response (22).

## PD-L1 Expression in RCC and Prognostic Significance

Tumor PD-L1 expression has not been found to be a marker of OS benefit in patients with aRCC treated with nivolumab (23). Although associations between tumor PD-L1 expression and improved outcomes have been observed with nivolumab in metastatic melanoma and non-small cell lung cancer (24), the predictive role of PD-L1 status on treatment outcomes remains to be determined. Many limitations have appeared with the use of PD-L1 expression as a potential biomarker for nivolumab activity. This is an area of ongoing research as there is currently a lack of identified biomarkers that reliably predict treatment benefit with immune checkpoint inhibitors (25).

## Nivolumab versus Everolimus Second-Line Study that Led to FDA Approval

In a phase II dose-ranging trial involving patients with metastatic RCC, the PD-1 inhibitor nivolumab was found to produce objective responses in 20–22% of patients with an OS ranging between 18.2 and 25.5 months. In the pivotal phase III study CheckMate 025, 821 patients with aRCC who received previous anti-angiogenic therapy were randomized to receive nivolumab 3 mg/kg every 2 weeks or everolimus 10 mg daily. Fifty percent of the patients had an intermediate prognosis and 15% a poor prognosis based on the Memorial Sloan Kettering Cancer Center risk assessment. In this study, the objective response rate (ORR) was greater with nivolumab (25% vs. 5%) and the OS was 25.0 vs. 19.6 months favoring nivolumab. Grade 3 or 4 treatment-related adverse events were lower in the nivolumab group (23). The results of this study led to its FDA approval in 2015.

## Adverse Events and their Management

Immune checkpoint inhibition is associated with a unique spectrum of side effects termed immune-related adverse events (irAEs), for example, endocrinopathies, diarrhea, colitis, hepatitis, pneumonitis, interstitial nephritis, and rash (26). These are typically transient, but occasionally can be moderate or severe. The incidence of irAEs can be unpredictable, and continuous vigilance for symptoms suggestive of irAEs is recommended. In general, many toxicities can be detected by routine blood work, and liver, kidney, and thyroid functions should be closely monitored. Management of irAEs can include either the use of corticosteroid immunosuppression or interruption of the checkpoint inhibitor. Further research is required to advance our understanding of the mechanisms underlying the development of these toxicities and improve upon current management strategies (27). In a phase I study, nivolumab was generally well tolerated, with 83% of patients developing adverse events but only 11% developing grade 3/4 toxicity (24). This favorable safety profile was later confirmed in the phase II and phase III trials (23, 28). The most common toxicity was fatigue, occurring in 33% of patients, followed by nausea (14%), diarrhea (12%), decreased appetite (12%), pruritus (14%), and rash (10%).

## Nivolumab Plus Ipilimumab in the Front Line

Escudier et al. (29) presented the findings from the phase III, open-label CheckMate 214 study at the ESMO 2017 Congress. In this trial, patients were randomized 1:1 to nivolumab (3 mg/kg) plus ipilimumab (1 mg/kg) every 3 weeks for four doses, followed by nivolumab, 3 mg/kg every 2 weeks; or to receive oral sunitinib, 50 mg a day for 4 weeks in 6-week cycles. Combined immunotherapy with nivolumab plus ipilimumab resulted in greater ORR and prolonged PFS when compared with sunitinib in intermediate- and poor-risk patients with previously untreated aRCC.

After 17.5 months of follow-up, ORR in intermediate- /poor-risk patients was 41.6% for the nivolumbab/ipilimumab combination compared with 26.5% for sunitinib. Around 9.4% of patients receiving the combination therapy achieved complete response compared with 1.2% of patients receiving sunitinib. Median PFS was 11.6 months for the nivolumab and ipilimumab combination versus 8.4 months with sunitinib.

Greater benefit was observed in patients with higher levels of PD-L1 expression at baseline. The ORR significantly favored nivolumab plus ipilimumab over sunitinib in intermediate- /poor-risk patients having a baseline PD-L1 expression ≥1% (58% vs. 25%, P = 0.0002). Median PFS was also significantly prolonged (22.8 months vs. 5.9 months, P < 0.0001). Patients with PD-L1 expression <1% did not benefit from the combination (HR, 1.00; P = 0.9670) (29).

## Future Directions

Checkmate 016 investigated nivolumab in association with VEGF inhibition with sunitinib or pazopanib. The ORR was 52% in the sunitinib arm and 45% in the pazopanib arm. Grade 3/4 treatment-related adverse events were observed in 82% of patients receiving nivolumab and sunitinib and in 70% of patients receiving nivolumab and pazopanib. Thirty-six percent of patients in the sunitinib arm and 25% in the pazopanib arm had to discontinue therapy. Most of the grade 3/4 adverse events were related to transaminitis/hepatotoxicity (30). These early studies have generated some enthusiasm and multiple combination trials of immunotherapy with or without VEGF-targeted therapy are ongoing.

## Conflicts of interest

RW Joseph is in the advisory/consulting boards for Bristol-Myers Squibb (BMS), Merck, Exelixis, Incyte, and Novartis. He has performed clinical trials with BMS, Merck, Roche, Amgen, X4P, and Syndax. The authors have no other relevant affiliations or financial involvement with any organization or entity with a financial interest in or financial conflict with the subject matter or materials discussed in the manuscript.
